# Xerophthalmia and Its Associated Factors among School-Age Children in Amba Giorgis Town, Northwest Ethiopia, 2018

**DOI:** 10.1155/2019/5130904

**Published:** 2019-11-22

**Authors:** Gizachew Tilahun Belete, Assefa Lake Fenta, Mohammed Seid Hussen

**Affiliations:** University of Gondar, College of Medicine and Health Science, School of Medicine, Department of Optometry, P.O.Box 196, Gondar, Ethiopia

## Abstract

**Introduction:**

Xerophthalmia is a general term applied to all the ocular manifestations from night blindness through complete corneal destruction (keratomalacia) due to vitamin A deficiency. Xerophthalmia is the main contributing factors for childhood blindness in developing countries. However, there is limited evidence that can implicate the current situation. This study aimed to determine the magnitude of xerophthalmia and associated factors among school-age children in Northwest Ethiopia.

**Methods:**

A community-based cross-sectional study was conducted on 490 children, age range of 6 to 12 years. The study participants were selected through systematic random sampling method. Data were collected using a pretested structured questionnaire and ophthalmic examination with different ophthalmic instruments. The analyzed result was summarized and presented using descriptive statistics. Binary logistic regression was used to determine the factors associated with xerophthalmia. Variables with a *p* value of <0.05 in the multivariable logistic regression analysis were considered as statistically significant.

**Results:**

A total of 484 study participants with a response rate of 98.8 were involved in this study, and their median age was 8 years with IQR of 4 years. The prevalence of xerophthalmia was 8.26% (95% CI: 5.8, 10.7). Family income less than 1000 Ethiopian birr (AOR = 4.65, 95% CI: 1.31, 16.4), presence of febrile illness (AOR = 2.8, 95% CI: 1.49, 6.11), poor consumption of fruits and vegetables (AOR = 3.18, 95% CI: 1.30, 7.80), and nonimmunized status (AOR = 3.43, 95% CI: 1.49, 7.89) were significantly associated with xerophthalmia.

**Conclusions and recommendations:**

The prevalence of xerophthalmia was high as compared to the World Health Organization criteria for public health significance. Factors identified for xerophthalmia in this study are low income, the poor dietary practice of fruits and vegetables, and the presence of febrile illness and not immunized. Hence, it is a public problem that needs attention.

## 1. Introduction

Xerophthalmia is the general term applied to all the ocular manifestations from night blindness through complete corneal destruction (keratomalacia) due to vitamin A deficiency [1]. It is the main contributing factors for childhood blindness in developing courtiers [2]. Around 250 million preschool children are at risk of vitamin A deficiency, mainly in developing countries [3–5]. Currently, it is estimated that there are about 1.5 million blind and 5 million visually disabled children worldwide due to the problem. About 350 thousand children become blind every year as a result of xerophthalmia [6].

Vitamin A deficiency affects growth, differentiation of epithelial tissues, and immune competence. On the eye, it results in night blindness, xerosis of the conjunctiva and cornea, and ultimately corneal ulceration and necrosis of the cornea [7].

The severity of xerophthalmia ranges from the mildest form, night blindness to ulceration and destruction of the cornea [6], eventually leading to blindness if left untreated [8–12]. The problem finally results in limitation of development, educational performance, and social and employment opportunities in the society [13]. Several shreds of evidence showed that insufficiently varied diets, poor maternal education, large family size, droughts, poverty, ignorance, faulty feeding habit, living in an endemic area, respiratory or diarrheal illness, and inadequate hygiene are positive factors for xerophthalmia [3, 13–17].

Different strategies were intended for the prevention and control of xerophthalmia globally, but the diseases are still the main cause of childhood blindness, especially in a low-income country [2, 18]. Childhood eye diseases like xerophthalmia are among the priorities of vision 2020 “the right to sight” [3, 19]. However, limited pieces of evidence implicated the situation, and they were institutional which might not reflect the burden of the problem in the community.

Researches done across different Asian countries showed that the prevalence of xerophthalmia ranges from 0.48% to 22.3% in different target populations [20–27].

A population-based survey conducted in Nigeria [28], Malawi [29], and Cameron [30] revealed that the prevalence of xerophthalmia ranges from 0.62% to 3.9%. Likewise, a community-based study performed in different regions of Sudan reported that the maximum prevalence of xerophthalmia was 8.8% [17, 31]. Community- and institution-based studies in different parts of Ethiopia showed that the prevalence of xerophthalmia extends from 0.48% to 20.8% [3, 32–39].

Different works of the literature find out various sociodemographic factors, socioeconomic factors, dietary habits (food fortification), vitamin A supplementation, medical disorders, health-related conditions, and immunization status had an association with xerophthalmia [10, 21, 34, 38–47].

Generally, xerophthalmia is the major cause of childhood blindness and public health problems in developing countries. Despite this, in most developing countries where xerophthalmia is endemic, people have incomplete knowledge concerning both the prevalence and the underlying associated factors. Therefore, this research aims to determine the magnitude and associated factors of xerophthalmia among school-age children.

## 2. Methods and Materials

### 2.1. Study Design, Source, and Sample Population

A community-based cross-sectional study with systematic random sampling technique was conducted among school-age children in Amba Giorgis town from April to May 2018. Amba Giorgis town is located in Northwest Ethiopia, which is around 780 km away from, Addis Ababa, the capital city of Ethiopia. The area is “Woyina dega” (weather condition between coldest and hottest). It has 3 urban “kebeles” (smallest administrative unit). According to the data obtained from the town administration statistical office, the total population in the town is 25,043. There were a total 2762 households with an assumption at least one child in each household. In the town, there are one kindergarten, 5 elementary schools, 2 high schools, and one preparatory school. There was also one health center and one primary hospital.

By using Epi info7.1 computer software and considering 95% confidence level, 80% power, and including 10% nonresponse rate, the final sample determined was 490.

### 2.2. Sampling Technique and Procedures

Systematic random sampling technique was used. To ensure representativeness, the sample was taken from all kebeles (smallest administrative unit) using the systematic random sampling method. In the three kebeles, there were 2762 households. With the assumption that there was at least one child in each household, the systematic random sampling method was used to select participating households proportionally by sampling fraction of 6 to get one child from each household. The lottery method was used to select the first household. If there were more than one child in a selected household, one child was selected randomly, and if no children in the selected household, the second household was considered.

### 2.3. Operational Definitions

#### 2.3.1. Xerophthalmia

The presence of one of the symptoms and clinical signs of xerophthalmia, i.e., night blindness and bitot's spot [11] with or without conjunctival xerosis, corneal xerosis with/without keratomalacia, and corneal scar bilaterally with the previous history of night blindness [11, 48].

#### 2.3.2. Febrile Illness

Having one or more of the following clinical findings like measles, diarrhea, respiratory infection, pneumonia, whooping cough, and attending health care.

### 2.4. Data Collection Procedures (Instrument and Personnel)

The standardized structured questionnaire was used to collect data regarding sociodemographic data, mother's/guardian's awareness on nutrition, and child dietary practice, factors leading protein-energy malnutrition, health-related problems of the child, immunization, and vitamin A capsule intake. Data recording format and clinical ocular examination and measurements were taken from the child with different ophthalmic instruments. A detailed ophthalmic examination was carried out by data collectors with strict adherence to standard methods and procedures. Any difficulties in case detection were discussed together. A pen torch and ×3  magnifiers loupe and direct ophthalmoscope were used to identify the clinical signs of xerophthalmia, such as bitot's spot, conjunctival xerosis, corneal xerosis, corneal ulceration, and corneal scar. But, the history of night blindness was confirmed by asking mothers/guardians using the local word for night blindness (‘*dafint*') whether a child faced any difficulty while playing or in identifying objects in dim light, especially at sunset. Six trained optometrists and one supervisor have participated in the data collection.

### 2.5. Data Processing and Analysis

The collected data were entered to Epi info 7.1 and was exported to SPSS version 20 for analysis. Descriptive statistics were used to present the result. The binary logistic regression model was fitted to determine the associated factors. The variables with a *p* value of <0.05 in the multivariable logistic regression analysis were considered as statistically significant.

## 3. Results

### 3.1. Sociodemographic Characteristics of the Study Participants

A total of 484 study participants were involved, with a response rate of 98.8%. The median age of the study participants was 8 years with an interquartile range of 4 years. About fifty percent, 244 (50.4%), of the study participants were between the age group of 6–8 years and 260 (53.7%) were females. Fifty-three percent of the household monthly income was below 1000 Ethiopian birr (Table 1).

### 3.2. Prevalence of Xerophthalmia

The prevalence of xerophthalmia was 8.3% [50], and the most observed clinical sign of xerophthalmia is bitot's spot followed by night blindness (Table 2).

### 3.3. Dietary Practices of Study Participants

Concerning feeding practice of children, most mothers/guardians, 381 (78.7%), fed animal products to their children and 373 (77.1%) give fruits and vegetables. However, 453 (93.6%) of children parents/caretakers have no vegetable garden (Table 3).

### 3.4. Health-Related Behaviors of Study Participants

Regarding the health-related behaviors of the study participants, 116 (24%) of the children had a history of febrile illness. The majority, 404 (83.3%), of the study participants used tape water for drinking, and 415 (85.7%) of children were immunized (Table 4).

### 3.5. Factors Associated with Xerophthalmia

The analysis output of binary logistic regression showed that mothers/guardians educational status, family size, family monthly income, feeding of animal product, feeding of fruits and vegetables, febrile illness, using tape water for drinking, using spring water for drinking, availability of household functional latrine, VA capsule intake for the last one year, and immunization status were associated with xerophthalmia. Whereas in the multivariable logistic regression analysis, low household monthly income, fruits and vegetable feeding practice, and presence of febrile illness and immunization were found to be independently associated with xerophthalmia (Table 5).

As indicated in the above table, study participants who had a household monthly income of between 1000 and 1500 Ethiopian birr were 4.65 times more likely to develop xerophthalmia as compared with those who had a household income of greater than 1500 Ethiopian birr (AOR = 4.65, 95% CI: 1.317, 16.4). Those study participants who had no dietary practice of fruits and vegetables for the last one month were 3.18 times more likely to develop xerophthalmia as compared with those who had fruits and vegetable dietary practice (AOR = 3.18, 95% CI: 1.30, 7.80).

Likewise, study participants who had a history of febrile illness for the last one year were 2.82 times more likely to develop xerophthalmia as compared to those who had no history of febrile illness (AOR = 2.82, 95% CI: 1.305, 6.11). Study participants who had no history of immunization were 3.41 times more likely to develop xerophthalmia as compared to those who had a history of immunization (AOR = 3.413, 95% CI: 1.491, 7.894).

## 4. Discussion

An understanding of the prevalence and the factors associated with xerophthalmia adds a piece of new knowledge about the condition, leading to better understanding and better strategies in the management of the condition. It is vitally important to realize that children with the sign of xerophthalmia are only the “tip of the iceberg” and there will be many other children in the community who are going to be xerophthalmic but seems to have completely normal eyes and vision.

In this study, the prevalence of xerophthalmia was 8.3% (95% CI: 5.8, 10.7). This finding is consistent with other studies conducted in Dembia District, Northwest Ethiopia [39], Wukiro, Northern Ethiopia [3], Arsi, Southeast Ethiopia [37], and South Sudan [31]. The possible explanation could be the study areas have a relatively similar sociocultural background and the method used was also similar. The working definition, study period, and the tool were almost similar that gives a consistent result [48]. But, the magnitude of xerophthalmia in this study is higher than reports in Asgede Tsimbla rural district of Northern Ethiopia [38], Jimma town, Ethiopia [36], Southern Ethiopia [33], northern Sudan [17], traditional Quran bordering school of Sudan [49], Nigeria [28], Malawi [29], Cameron [30], Southeast Asia [50], Nepal [22], Philippines [23], northern Bangladesh [24], west java, Indonesia [25], Thailand [26], Cambodia [27], Yemen [51] and Ahmadabad, India [20]. This discrepancy might be due to the sociocultural variation among the study populations. The other source of discrepancy might be due to differences of operational definition in which most studies used the two clinical sign of xerophthalmia, i.e., night blindness and bitot's spot as diagnostic criteria whereas in this study the diagnosis of xerophthalmia is based on all the clinical sign of xerophthalmia except conjunctival xerosis that cannot always correlate with xerophthalmia. On the other hand, studies performed in the Hararge region of Ethiopia [32], Bushulo, rural south Ethiopia [35], and Fadis Oromia regional state, Ethiopia [34] showed a higher prevalence of xerophthalmia. This discrepancy might be due to study designs and target population. For instance, in the Hararge region of Ethiopia, the study surveyed throughout the regions which include rural areas that might increase the prevalence, and the study at Bushulo health center in rural southern Ethiopia was an institutional-based study and the target population was malnourished children.

Another issue for this discrepancy could be regional variations in sociocultural, socioeconomic status, and health-seeking behavior.

Regarding factors related with xerophthalmia, study participants who had a household monthly income between 1000 and 1500 birr were 4.65 times more likely to develop xerophthalmia as compared with those having household monthly income greater than 1500 Ethiopian birr. This result is supported by a study performed in north Sudan [17] and in Aligarh District, Uttar Pradesh [43]. This is because shortages of food especially vitamin A-rich sourced food are the commonest cause of xerophthalmia, and if family income is low, mothers or guardians cannot afford to purchase and access VA rich foods for their children. Generally, xerophthalmia is a disease related to low socioeconomic status and poverty.

Those children who had no dietary practice of fruits and vegetables for the last one month were 3.18 times more likely to be xerophthalmic as compared to those who had adequate dietary practice of fruits and vegetables. This finding is also in line with a study conducted in the Republic of Kiribati [41]. Many studies performed in different countries strongly support that fruits and vegetables consumptions are vital to alleviating xerophthalmia [11, 16, 44, 52, 53]. This finding has serious implications particularly when one considers the fact that plant sources are the major sources of vitamin A in low-income communities. Probably, the most common cause of vitamin A deficiency in developing countries is inadequate intake of vitamin A-containing foods. Vitamin A-rich source prevents xerophthalmia and decreases susceptibility to infection.

Those children who had a febrile illness for the last one year were 2.82 times more likely to be xerophthalmic as compared with those children who had no febrile illness. This finding is in line with a study performed in Aligarh District, Uttar Pradesh, India [43], and Cambodia [27] that shows children with diarrhea were independently associated with xerophthalmia. The reason behind might be that febrile illness like diarrhea affects vitamin A status by increasing loss of nutrients substantially. Studies also show intestinal parasitosis such as giardia, ascaris, and hookworms reduce the absorption of nutrients [17, 54]. Respiratory tract infections also compromise the dietary absorption of vitamin A.

Those children who had no history of immunization were 3.43 times more likely to be xerophthalmic as compared with those children who had a history of immunization. This finding is supported by a study performed in Jima, Ethiopia [55] and Aligarh District, Uttar Pradesh, India. The importance of immunization in preventing xerophthalmia was highlighted by the finding of this study. Immunization has the ability to provide protecting infectious diseases.

This study implies xerophthalmia was public health significance for school children of this society. In the long term, sustainable control must be achieved by increasing the production and consumption of foods rich in vitamin A and carotene by at-risk populations. Other methods include treating febrile illness accordingly, often consisting of high doses of vitamin A every four to six months and fortification of foods, and nutrition education must be handled.

## 5. Conclusion

In this study, the prevalence of xerophthalmia in school-age children was higher as compared to the WHO cutoff point for public health significance. Low household income, inadequate dietary practice of fruits and vegetables, presence of febrile illness, and no history of immunizations were the factors that were positively associated with xerophthalmia.

## Figures and Tables

**Table 1:** Sociodemographic characteristic of the study participants in Amba Giorgis town, Northwest Ethiopia.

Variables	Frequency (*n* = 484)	Percentage
Sex		
Female	260	53.7
Male	224	46.3

Age (in year)		
6–8	244	50.4
9–10	125	25.8
11–12	115	23.8

Mother's/guardian's educational status		
Uneducated	303	62.6
Educated	181	37.4

Family size		
1–4	151	31.2
>4	333	68.8

Mother's/guardian's occupation		
House wife	297	61.4
Labor worker	70	14.5
Governmental	65	13.4
Merchant	40	8.3
Others^*∗*^	12	2.5

Family/household monthly income (birr)		
<1000	225	52.7
1000–1500	56	11.6
>1500	173	35.7

^*∗*^Pensioner, beggar, and weaver.

**Table 2:** Clinical presentation of xerophthalmia among school-age children of Amba Giorgis town, Northwest Ethiopia.

Variables	Frequency (*n* = 484)	Percent
Bitot's spot	22	4.5
Night blindness	15	3.1
Corneal xerosis	2	0.4
Corneal scar	1	0.2

**Table 3:** Dietary practices of study participants living in Amba Giorgis town, Northwest Ethiopia.

Variables	Frequency	Percent
Feed animal products (*n* = 484)		
Yes	381	78.7
No	103	21.3

Frequency of feeding animal products (*n* = 381)		
Once a week	147	38.6
Twice a week	123	32.3
Once a month	104	27.3
Everyday	7	1.8

Feed fruits and vegetables (*n* = 484)		
Yes	373	77.1
No	111	22.9

Frequency feeding fruit and vegetable (*n* = 373)		
Once a week	191	51.1
Twice a week	92	24.6
Once a month	73	19.5
Everyday	18	4.8

Presence of vegetable garden (*n* = 484)		
No	453	93.6
Yes	31	6.4

**Table 4:** Health-related problems of the study participants living in Amba Giorgis town, Northwest Ethiopia (*n* = 484).

Variables	Frequency	Percent
Febrile illness in the last one year		
No	368	76
Yes	116	24

Using tape water for drinking		
Yes	404	83.3
No	80	16.5

Using pond water for drinking		
No	415	85.7
Yes	69	14.3

Using spring water for drinking		
No	366	75.6
Yes	118	24.4

Functional house hold latrine availability		
Yes	339	70
No	145	30

VA capsule intake for the last 1 year		
Yes	267	55.2
No	217	44.8

Immunization status		
Immunized	415	85.7
Nonimmunized	69	14.3

**Table 5:** Factors associated with xerophthalmia of study participants living in Amba Giorgis town, Northwest Ethiopia (*n* = 484).

Xerophthalmia				
Variable	Yes	No	COR (95% CI)	AOR (95% CI)
Age (in year)				
6–8	18	226	1.00	
9–10	10	115	1.1 (0.488, 2.44)	
11–12	12	103	1.46 (0.680, 3.15)	

Mother's/guardian's educational status				
Educated	7	174	1.00	1.00
Uneducated	33	270	3.038 (1.32, 7.0)	1.336 (0.523, 3.566)

Family size				
1–4	7	144	1.00	1.00
>4	33	300	2.263 (0.977, 5.239)	1.62 (0.632, 4.156)

Family/household income (birr)				
1000	26	229	3.815 (1.44, 10.14)	1.665 (0.555, 4.99)
1000–1500	9	47 6.434 (2.059, 20.119)	**4.648 (1.317, 16.4)** ^**∗**^	
>1500	5	168	1.00	1.00

Feed animal products				
No	18	85	3.456 (1.775, 6.728)	1.124 (0.476, 2.66)
Yes	22	359	1.00	1.00

Feed fruits and vegetables				
No	24	87 6.155 (3.15, 12.1)	**3.18 (1.30, 7.8 0)** ^**∗**^	
Yes	16	357	1.00	1.00

Vegetable garden				
No	36	417	0.583 (0.193, 1.757)	
Yes	4	27	1.00	

Febrile illness				
No	22	346	1.00	
Yes	18	98	2.889 (1.490, 5.601)	**2.824 (1.305, 6.11)** ^**∗**^

Using tap water for drinking				
Yes	16	64	3.958 (1.994, 7.858)	1.1 (0.362, 3.22)
No	24	380	1.00	1.00

Using pond water for drinking				
No	33	382	1.00	
Yes	7	62	1.307 (0.554, 3.1)	

Using spring water for drinking				
No	21	345	1.00	1.00
Yes	19	99	3.153 (1.630, 6.1)	1.248 (0.474, 3.286)

Latrine				
No	21	124	2.852 (1.483, 5.487)	1.1 (0.463, 2.59)
Yes	19	320	1.00	1.00

VA capsule				
No	31	236	3.036 (1.412, 6.525)	1.342 (0.534, 3.369)
Yes	9	208	1.00	1.00

Immunization				
No	16	53	4.918 (2.456, 9.851)	3.431 (1.491, 7.894)^**∗**^
Yes	24	391	1.00	1.00

^**∗**^ *p* value < 0.05(significant); COR: crude odds ratio; AOR: adjusted odds ratio.

## Data Availability

The data sets generated and analyzed for the current study are available from the corresponding author upon reasonable request.
